# Self-Powered Detection of Glucose by Enzymatic Glucose/Oxygen Fuel Cells on Printed Circuit Boards

**DOI:** 10.1021/acsami.1c02747

**Published:** 2021-05-26

**Authors:** Carla Gonzalez-Solino, Elena Bernalte, Clara Bayona Royo, Richard Bennett, Dónal Leech, Mirella Di Lorenzo

**Affiliations:** † Department of Chemical Engineering, University of Bath, Bath BA2 7AY, U.K.; ‡ Centre for Biosensors, Bioelectronics and Biodevices (C3Bio), University of Bath, Bath BA2 7AY, U.K.; § School of Chemistry & Ryan Institute, National University of Ireland Galway, University Road, Galway H91 TK33, Ireland

**Keywords:** enzymatic fuel cell, printed circuit board, glucose monitoring, self-powered detection, highly porous gold

## Abstract

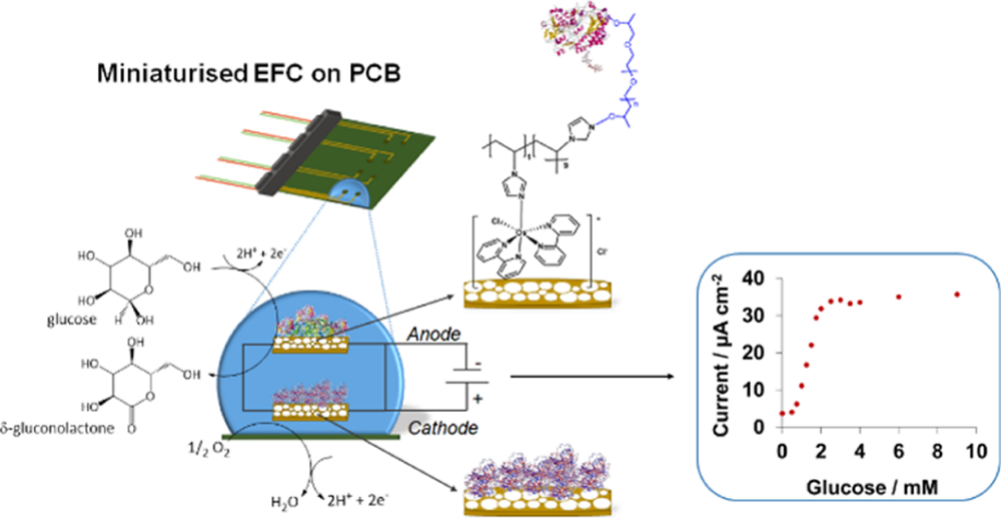

Monitoring glucose levels in physiological fluids can help prevent severe complications associated with hypo- and hyper-glycemic events. Current glucose-monitoring systems require a three-electrode setup and a power source to function, which can hamper the system miniaturization to the patient discomfort. Enzymatic fuel cells (EFCs) offer the opportunity to develop self-powered and minimally invasive glucose sensors by eliminating the need for an external power source. Nevertheless, practical applications demand for cost-effective and mass-manufacturable EFCs compatible with integration strategies. In this study, we explore for the first time the use of gold electrodes on a printed circuit board (PCB) for the development of an EFC and demonstrate its application in saliva. To increase the specific surface area, the PCB gold-plated electrodes were modified with porous gold films. At the anode, glucose oxidase is immobilized with an osmium redox polymer that serves as an electron-transfer mediator. At the cathode, bilirubin oxidase is adsorbed onto the porous gold surface with a blocking agent that prevents parasitic reactions while maintaining the enzyme catalytic activity. The resulting EFC showed a linear response to glucose in phosphate buffer within the range 50 μM to 1 mM, with a sensitivity of 14.13 μA cm^–2^ mM^–1^. The sensor was further characterized in saliva, showing the linear range of detection of 0.75 to 2 mM, which is within the physiological range, and sensitivity of 21.5 μA cm^–2^ mM^–1^. Overall, this work demonstrates that PCBs are suitable platforms for EFCs, paving the way for the development of fully integrated systems in a seamless and miniaturized device.

## 1 Introduction

Diabetes is a metabolic condition characterized by the inability to control glucose levels in blood, which affects more than 400 million people worldwide.^1^ Uncontrolled glycemia leads to severe health complications, such as nerve injuries or blindness.^2^ The incidence of hypo- or hyper-glycemic events can be prevented through continuous monitoring of glucose levels in blood. In this regard, enzymatic fuel cells (EFCs) have demonstrated great potential for glucose sensing.^3^ In an EFC, the current is generated by coupling the oxidation of glucose to the reduction of oxygen at two separate electrodes that are connected through an external circuit. When a load is applied to such a circuit, the system produces a continuous current that is proportional to the concentration of glucose.^4^ No external power source is required which, compared to other types of sensors, drastically simplifies the electronics required and allows miniature designs.

Glucose oxidation at the anode is usually catalyzed by the enzyme glucose oxidase (GOx), whereas bilirubin oxidase (BOD) can be used for oxygen reduction at the cathode. Although BOD can accept electrons directly from the electrode, GOx requires a redox mediator to facilitate electron transfer.^5^ Osmium-based redox polymers have demonstrated efficient wiring of GOx to electrode surfaces allowing fast and effective electron transfer.^6^ Moreover, the use of nanostructured electrodes helps maximize the current and power densities by allowing high-enzyme loadings compatible with small geometric areas of the electrodes, which are required for miniature devices.^7^ In particular, nanoporous and highly porous gold (hPG) have been widely tested for the immobilization of enzymes.^8^ Nonetheless, nanostructured gold electrodes are highly reactive toward small molecules present in physiological fluids.^9^ When this type of electrodes is used for EFCs, a poor surface coverage by the enzyme could, therefore, interfere with the enzymatic processes, eventually affecting the output power.

For wearable applications, the electrodes of the EFC must be integrated into a single and miniaturized platform to generate minimally invasive devices that can be smoothly blended with the patient’s everyday activities. Prototypes of minimally invasive EFCs have been fabricated on textiles,^10^ paper,^11^ or flexible polymers.^12^ The electrodes of these devices are usually fabricated by either screen printing^13^ or 3D printing.^14^ As an alternative, the printed circuit board (PCB) industry offers rapid, long-standing, and standardized manufacturing of electronic circuits that can be exploited for electrochemical sensing and fuel cells.^15,16^ The PCB technology offers great design flexibility, allowing customization for tailored applications. The manufacturing cost of PCBs ($0.20 per cm^2^) is comparable to those of paper and polymer-based platforms.^17^ PCBs, however, have the advantages of being easily integrated with electronics and microfluidic modules,^16^ thus reducing the overall size of the device as well as generating solutions compatible with mass production.

In this work, we present the first EFC on a PCB and demonstrate its potential use for the self-powered detection of glucose in artificial saliva. Saliva offers great potential for the non-invasive detection of analytes due to its easy accessibility and good correlation with glucose levels in blood.^18^ Recently, several EFCs operating in saliva have been reported,^19,20^ but the self-powered detection of glucose is not yet demonstrated. First, the effect of increasing the electrochemical surface area (ESA) of PCB-based gold-plated (Au) electrodes with hPG films is investigated to enhance the overall electrochemical performance of the immobilized enzymes. Then, the resulting EFC is operated in phosphate buffer and artificial saliva to demonstrate its potential application as a self-powered diagnostic device in physiological fluids.

## 2 Materials and Methods

### 2.1 Reagents

All chemicals were of analytical grade and used as received unless otherwise specified. Hydrogen tetrachloroaurate(III), calcium chloride, glucose, and potassium chloride were obtained from Fisher. Ammonium chloride, citric acid, magnesium sulfate, polyethylene glycol diglycidyl ether (PEGDGE), potassium thiocyanate, sodium chloride, sodium phosphate dibasic, sodium phosphate monobasic, and sulfuric acid were purchased from Sigma-Aldrich.

The enzyme GOx from *Aspergillus niger* Type X-S (100 to 250 U mg^–1^) was purchased from Sigma-Aldrich. BOD from *Myrothecium verrucaria* (1.2 U mg^–1^) was purchased from Amano Enzymes, Japan.

The osmium redox polymer used, [Os(2,2′-bipyridine)_2_(polyvinylimidazole)_10_Cl]Cl denoted OsPVI, was synthesized using literature procedures.^21,22^

All aqueous solutions were prepared with ultrapure water (18.2 MΩ cm^–1^) from a Milli-Q water system (Merck Millipore, UK). Phosphate buffer (PB) at a concentration of 0.1 M and pH 7.4 was prepared by dissolving sodium phosphate monobasic and sodium phosphate dibasic in Milli-Q water. A stock solution of 2 M glucose was prepared in PB and left overnight to allow the mutarotation from α- to β-monomer.^23^ The glucose solution was kept at 4 °C and used within 2 weeks.

Synthetic saliva was prepared as previously described by Kim et al.^24^ and kept in dark and at room temperature.

### 2.2 Electrodes and Apparatuses

The PCBs were designed with a computer-aided design software, Altium Designer, and commercially manufactured in a standard PCB manufacturing facility (Lyncolec Ltd., UK). Each PCB comprises two circular electrodes, with an area of 1.54 mm^2^ each, separated from each other by a 0.85 cm gap. All experiments were performed with an Autolab potentiostat (PGSTAT302N), and controlled by Nova software (Metrohm, UK). The PCB was connected to the potentiostat through a peripheral component interconnect purchased from RS Components (UK). The electrodes were individually characterized in a three-electrode setup by using refillable miniature Ag/AgCl (3 M KCl) as the reference electrode (ET073, Green Leaf Scientific, Ireland) and a Pt wire (0.5 mm diameter, Alfa Aesar, UK) as the counter electrode.

### 2.3 Deposition of hPG onto PCB Gold-Plated Electrodes

Prior to use, Au electrodes were activated in 0.05 M H_2_SO_4_ by cycling the potential from −0.5 to 1.6 V versus Ag/AgCl for 12 cycles, at a scan rate of 100 mV s^–1^. The PCB Au electrodes were functionalized with a film of hPG to increase the surface area. The hPG film was deposited via the dynamic hydrogen bubbling template as previously described by our group.^15^ The ESA of both the Au and hPG/Au electrodes was calculated from the reduction peak of gold oxide in sulfuric acid, as previously described.^25^

The Au and hPG/Au electrodes were also characterized by scanning electron microscopy (SEM) with the use of a JEOL JSM-6480LV SEM. Figure S1 shows the surface morphology of the two electrodes.

### 2.4 Enzyme Immobilization onto hPG/Au Electrodes on PCB

GOx, cross-linked with an osmium-based polymer [Os(2,2′-bipyridine)_2_ (polyvinylimidazole)_10_Cl]Cl (OsPVI) using PEGDGE, was used as the biocatalyst at the anode of the EFC. A stock solution was prepared by mixing 8 μL of 5 mg mL^–1^ osmium polymer with 8 μL of 5 mg mL^–1^ GOx and 4 μL of 15 mg mL^–1^ PEGDGE solution. The amounts of enzyme, osmium-based redox polymer, and PEGDGE solution were chosen based on previous research.^26^ Two microliters of this stock solution was then drop-cast onto the hPG/Au electrodes and allowed to evaporate at room temperature for 1 h. The surface coverage of the Os polymer onto the hPG/Au electrode was estimated according to eq 1
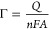
1where *Q* (C) is the charge required for either the reduction or oxidation of the Os polymer; n is the number of electrons involved; *F* (96,486 C mol^–1^) is the Faraday constant, and A (cm^2^) is the geometric surface area of the electrode. The charge of the electrodes was calculated by cycling the potential of the electrode in 0.1 M phosphate buffer, pH 7.4, at a scan rate of 10 mV s^–1^ versus Ag/AgCl.

**Figure 1:**
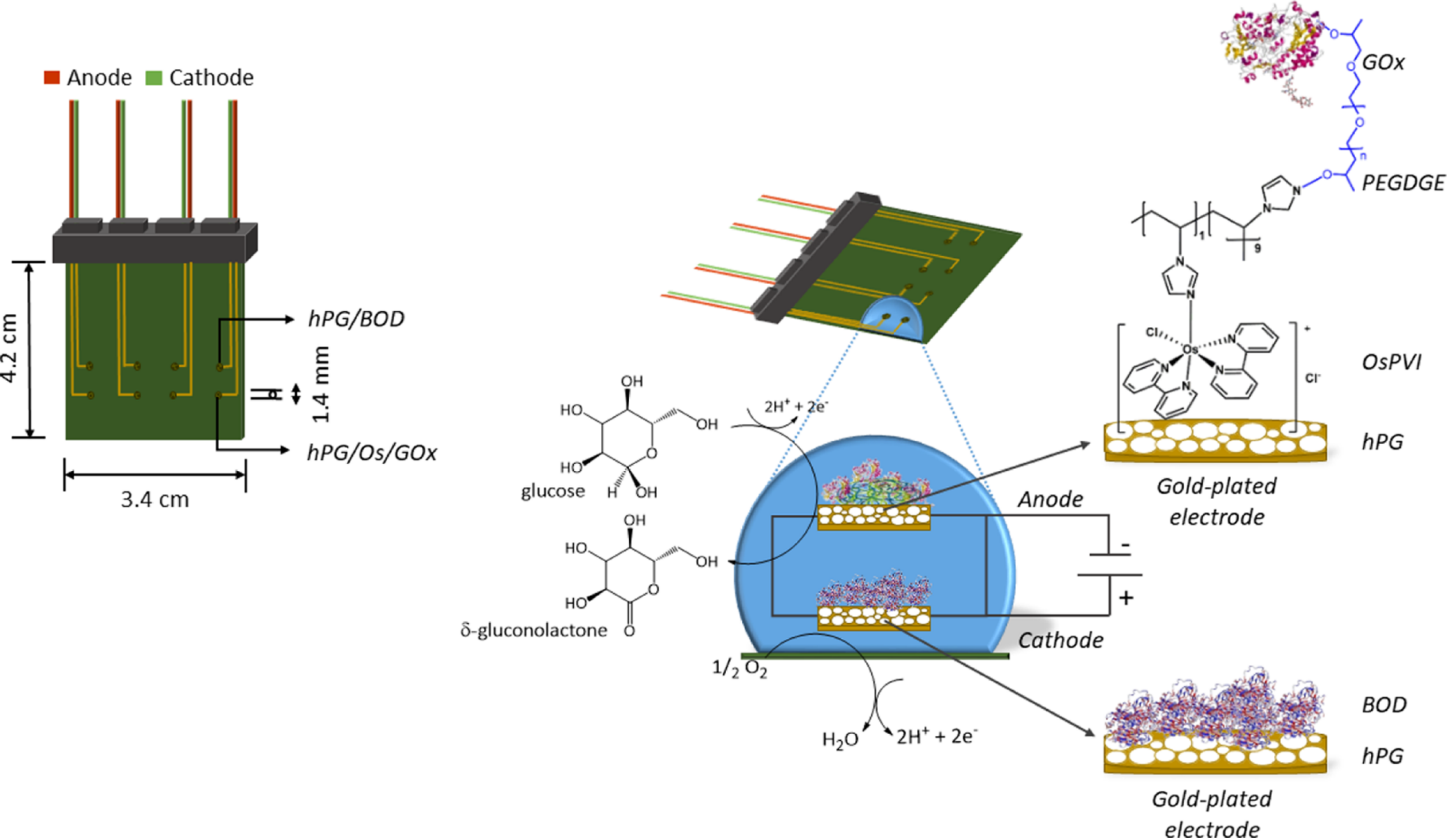
Schematic of the EFC on PCB, showing the GOx/Os/hPG/Au anode and the SB/BOD/hPG/Au cathode, along with the respective electrochemical reactions involved.

The biocathode was prepared by drop-casting 4 μL of a 1 mg mL^–1^ BOD solution in 0.1 M PB, pH 7.4, onto the hPG/Au electrode surface and let it evaporate at room temperature for 1 hour. To prevent glucose oxidation at the cathode, the StartingBlock (SB) solution (Thermo Fisher, UK), was used. In this case, the BOD/hPG/Au electrodes were incubated with SB for 30 min at room temperature, washed with Milli-Q water to remove any unbound molecules, and stored at 4 °C prior to use. Figure 1 shows the developed EFC on a PCB, including the schematics of the electrodes and the reactions involved.

### 2.5 Bioanode and Biocathode Characterization

Both bioelectrodes were characterized in a three-electrode cell at room temperature in 0.1 M phosphate buffer, pH 7.4, unless otherwise specified. The biocathode was characterized by linear sweep voltammetry (LSV) at a scan rate of 5 mV s^–1^ in an oxygen-saturated buffer with and without 6 mM glucose. The bioanode was characterized by cyclic voltammetry (CV) at a scan rate of 5 mV s^–1^ in the presence and in the absence of 6 mM glucose in an air-saturated buffer to determine the catalytic activity of the electrode toward glucose. The sensitivity of the electrode toward glucose was further characterized by chronoamperometry at an applied potential of +0.22 V versus Ag/AgCl (3 M KCl) at increasing concentrations of glucose, spanning from 50 to 100 mM.

### 2.6 Fuel Cell Characterization

The EFC was connected to a PicoData Logger ADC-24 (Pico Technology, UK) to monitor the cell potential over time. First, the EFC was left in an open circuit potential (OCP) in 6 mM glucose in 0.1 M phosphate buffer, pH 7.4, until reaching the steady state. Afterward, polarization tests were performed by varying the external resistance applied to the system, from 10 to 1000 Ω, with a Cropico resistor box (RS Components, UK). The power curves were obtained from the polarization curves, and the power was calculated using eq 2
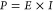
2where *P* (W) is the power output; *E* (V) is the cell voltage; and *I* (A) is the current drawn from the fuel cell. The internal resistance (*R*_int_) of the EFC was calculated from the linear fit of the Ohmic region of each polarization curve (*R*_int_ = Δ*V*/Δ*I*). The power and current densities are normalized to the geometrical surface area of the bioanode (1.54 mm^2^) throughout this study.

The calibration curves for the self-powered detection of glucose were obtained by continuously monitoring the current generated by the EFC upon increasing the concentrations of glucose at the optimal external resistance. The current output was measured starting from a blank solution, either phosphate buffer or artificial saliva, containing no sugar and progressively adding glucose. After each addition, the EFC was left to reach a steady value of output current before adding the next concentration. The steady-state output current values obtained for each concentration of glucose were used as data points for the calibration curve.

## 3 Results and Discussion

### 3.1 Characterization of the Biocathode

**Figure 2:**
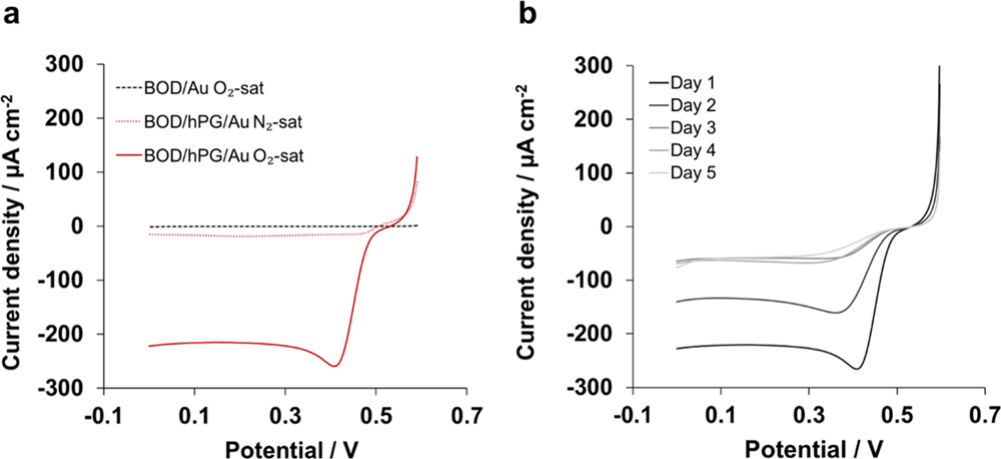
Investigation of the biocathode performance via LSV at a scan rate of 5 mV s^–1^ in 0.1 M phosphate buffer, pH 7.4. (a) Comparison between BOD/Au and BOD/hPG/Au electrodes and (b) stability of the BOD/hPG/Au electrode over 5 days after saturating the solution with O_2_. The measurements were performed once a day.

First, the electrocatalytic activity of the biocathode was investigated. BOD is commonly exploited in the development of EFCs because of its ability for direct electron transfer and high stability under physiological conditions.^27^Figure 2 shows the enhancement of the electrocatalytic activity of the BOD/hPG electrodes compared to BOD/Au and its stability over time in oxygen-saturated solutions. The stability of the electrode was estimated by measuring the decay in the oxygen reduction reaction (ORR) at the BOD/hPG. The ORR current was measured once per day, and the electrodes were covered with a drop of phosphate buffer, pH 7.4, and kept in the fridge in-between measurements.

In agreement with previous reports, no catalytic reduction of oxygen was observed for the BOD immobilized onto the planar PCB Au electrodes,^28^ as shown in Figure 2a. In contrast, the *I–V* response of the BOD/hPG/Au electrode shows a reducing current starting at a potential of +0.49 V versus Ag/AgCl, which is in good agreement with reported values for BOD adsorbed onto nanoporous gold.^29^ This potential for the ORR shows a strong linear dependence on the pH of the electrolyte, with a slope of 40 mV/pH (*R*^2^ = 0.98) for pH values between 3 and 8, as shown in Figure S2a. This behavior approximates to a 1e^–^/1H^+^ process, as previously described for BOD.^30^ The ORR reaches a maximum current density of 282 ± 45 μA cm^–2^ (*n* = 4) at a potential of approximately +0.40 V versus Ag/AgCl. The BOD/hPG/Au shows similar current densities as previously reported electrodes based on nanoporous gold.^28^ As control, the ORR was studied in the absence of BOD, and no catalytic activity was observed within the potential window tested (Figure S2b). Likewise, no current response was observed when BOD/hPG/Au was tested in N_2_-saturated phosphate buffer (Figure 2a), confirming that BOD is successfully immobilized onto the hPG/Au cathode and is active toward oxygen.

Unspecific adsorption of enzymes usually results in lower stability of the bioelectrodes.^7^ Therefore, the stability of the biocathode was investigated by monitoring the ORR over 5 days (Figure 2b). As is shown, a decrease in the catalytic current is observed over the first 3 days with a total reduction of 41% in day 2 compared to day 1, which is consistent with previous studies reported on adsorbed BOD.^28,31^ No further decrease in the current peak was observed in the following days.

**Figure 3:**
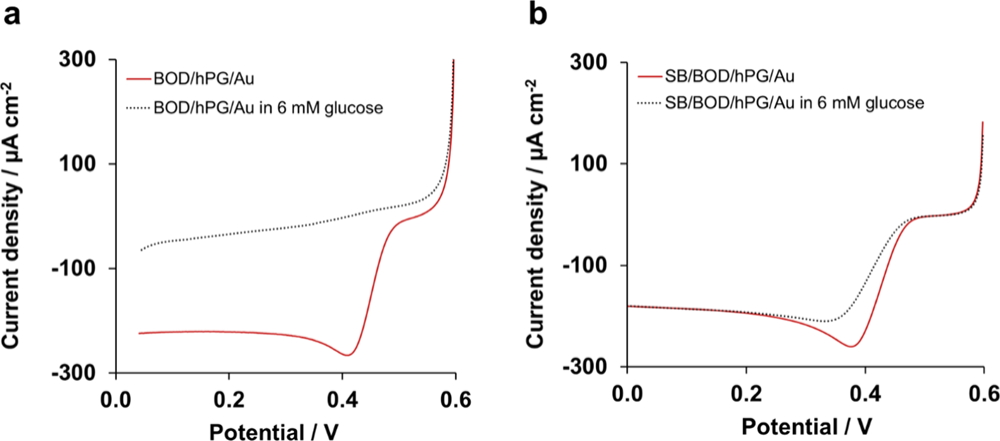
Catalytic response of the biocathode to glucose in the (a) absence and (b) presence of SB. The tests were performed in an O_2_-saturated 0.1 M phosphate buffer, pH 7.4. The graphs represent just one replicate.

Further characterization of the biocathodes in the presence of oxygen-saturated PB containing 6 mM glucose (Figure 3a) revealed a small oxidative current at +0.4 V versus Ag/AgCl. This oxidative process suggests poor covering of BOD onto the electrode surface leading to glucose oxidation by hPG, as a result of its high catalytic activity toward saccharides.^9^ In fact, the hPG/Au electrode, not functionalized with BOD, shows a sharp increase in current at a potential of +0.3 versus Ag/AgCl in the presence of glucose (Figure S3). Consequently, the use of a blocking buffer was investigated to cover the exposed hPG after BOD immobilization and avoid glucose oxidation. Blocking buffers, such as SB or Tris buffer solutions,^32^ or the use of inert proteins, such as bovine serum albumin,^33^ are commonly used in biosensor development to prevent unspecific binding. In this study, the addition of a blocking buffer (SB) on the electrochemical performance of the BOD/hPG/Au electrode was tested.

The electrode SB/BOD/hPG/Au did not show any response to glucose and generated a maximum current of 264 ± 30 μA cm^–2^ (*n* = 4) in an O_2_-saturated buffer containing 6 mM glucose with an onset potential of +0.47 V, Figure 3b. Therefore, these results confirm that the SB is effective in protecting the biocathode from glucose interference without affecting its catalytic activity toward oxygen.

### 3.2 Characterization of the Bioanode

Contrary to BOD, GOx is unable to directly transfer electrons. Therefore, GOx is usually immobilized with a redox mediator, such as osmium polymers or ferrocene derivatives.^6^ Combining redox mediators with highly porous electrodes can enhance electron transfer at the anode and consequently the output power. To highlight the benefit of combining porous structures and redox polymers, Figure S4 compares the currents obtained with the planar Au versus hPG/Au electrode after being modified with the osmium polymer. The *E*_1/2_ was very similar for both electrodes at approximately 200 mV versus Ag/AgCl. This half-wave potential is in agreement with previously reported values for the Os (II/III) redox couple at gold electrodes^34^ and nanoporous gold electrodes.^35^ The Os/hPG/Au electrode generated higher current outputs compared to Os/Au, with peak current values of 124 and 40 nA, respectively, in the absence of glucose and at a scan rate of 10 mV s^–1^. This result can be explained by the higher osmium loading for the hPG/Au electrodes, 1.59 ± 0.13 nmol cm^–2^, compared to the 0.64 ± 0.17 nmol cm^–2^ obtained for the Os/Au electrodes, which is a consequence of the larger specific surface area of hPG films, approximately 140 times higher than those of the PCB Au electrodes, as revealed by their ESAs (1.96 cm^2^ vs 0.01 cm^2^ for the hPG/Au and Au electrodes, respectively). Using the porous gold structure can therefore develop high-current-density FCs.

**Figure 4:**
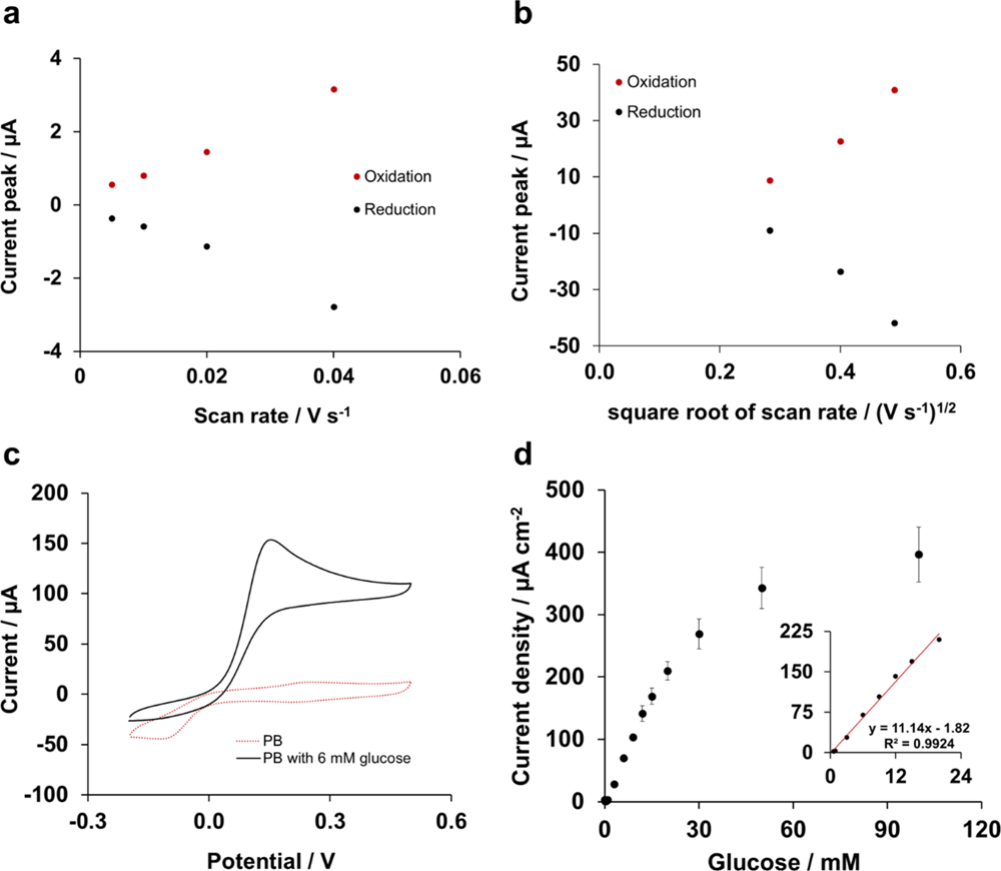
Characterization of the bioanode in 0.1 M phosphate buffer, pH 7.4. (a) Scan rate study for scan rate values lower than 40 mV s^–1^ and (b) scan rate study for scan rate values higher than 40 mV s^–1^. (c) Cyclic voltammograms of the bioanode GOx/Os/hPG/Au in air-equilibrated 0.1 M phosphate buffer, pH 7.4 in the presence and absence of 6 mM glucose. The CV curves were recorded versus Ag/AgCl at a scan rate of 5 mV s^–1^ and represent the *I*–*V* response for one independent electrode. (d) Chronoamperometric response at an applied potential of +0.22 V versus Ag/AgCl of the bioanode GOx/Os/hPG/Au to glucose (50 μM to 100 mM) in 0.1 M phosphate buffer, pH 7.4 (*y* = 11.14*x* −1.82, *R*^2^ = 0.9924). The inset corresponds to the linear range of the bioanode. Error bars refer to the standard error (*n* = 3).

The scan rate study of Os/hPG/Au revealed a linear dependence between the peak current and the scan rate at values lower than 40 mV s^–1^ (Figure 4a), which is typical of surface-confined processes. At higher scan rates, however, the peak currents showed a linear dependence on the square root of the scan rate (Figure 4b), which is associated with the diffusion-controlled process within the film.^36^

The catalytic activity toward glucose of the enzymatic electrode (GOx/Os/hPG/Au) was subsequently investigated by CV and chronoamperometry in phosphate buffer (Figure 4c,d). In the presence of 6 mM glucose, the electrode showed a catalytic current with an onset potential of +0.05 V and a maximum peak current density of 154 μA cm^–2^ at a voltage of approximately +0.15 V versus Ag/AgCl. As is shown, no current response was observed in the absence of glucose. The chronoamperometric response at an applied potential of +0.22 V versus Ag/AgCl (Figure 4d) of the GOx/Os/hPG/Au electrode showed a linear response up to 20 mM, with a limit of detection of 314 μM and a sensitivity of 11.2 μA mM^–1^ cm^–2^.

### 3.3 Characterization of the EFC

**Figure 5:**
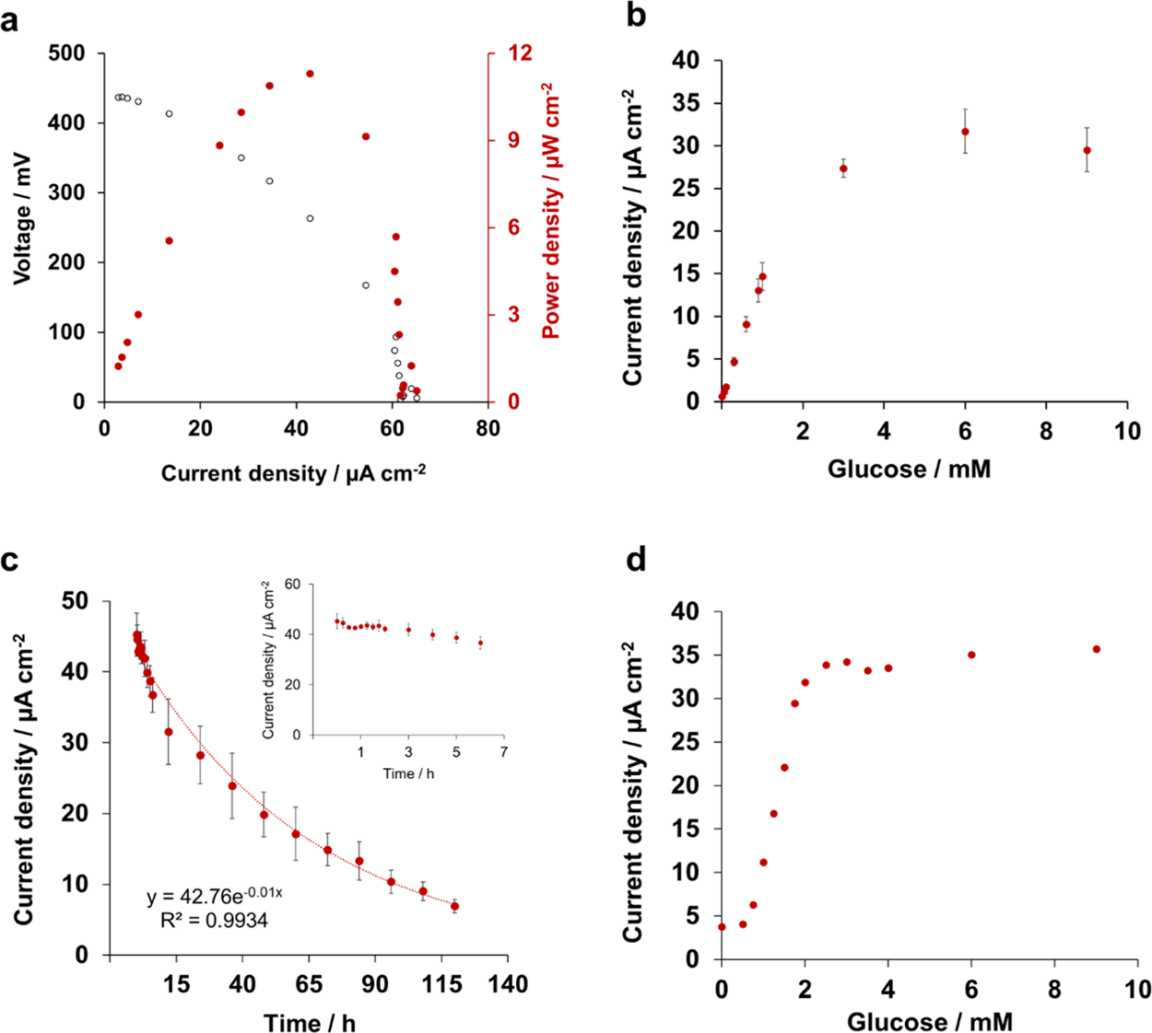
Characterization of the EFC: (a) polarization and power curve of the fuel cell in air-saturated phosphate buffer containing 6 mM glucose; (b) output current density under increasing concentrations of glucose in phosphate buffer; (c) continuous current output over time in phosphate buffer containing 6 mM glucose (inset shows the output current over the first 7 h of operation); and (d) output current density under increasing concentrations of glucose in artificial saliva. Error bars refer to the standard error (*n* = 3).

The performance of the EFC was evaluated in air-saturated phosphate buffer containing 6 mM glucose. The fuel cell generated an open circuit potential of 413 ± 13 mV (*n* = 3) that corresponds to the difference between the onset potentials for the reactions occurring at the cathode and the anode and aligns with previously reported glucose fuel cells.^37^ The fuel cell was subjected to a series of external resistance loads to obtain the polarization curve and the power-density profile of the fuel cell (Figure 5a). The polarization curve shows a steady decrease in voltage with a short-circuit-current density of ca. 60 μA cm^–2^. The power-density profile shows a maximum power density of 9.6 ± 1.4 μW cm^–2^ (*n* = 3). Such power output is limited by the high Ohmic and mass transport limitations that result in a high internal resistance of 330 kΩ. Despite this high internal resistance, the power output generated in this study aligns with previously reported glucose/oxygen fuel cells at similar oxygen and glucose concentrations.^38^

### 3.4 Self-Powered Detection of Glucose

The current output generated by the EFC is proportional to the concentration of glucose, allowing the self-powered detection of glucose. As shown in Figure 5b, the output current increased after each addition of glucose up to a concentration of 1 mM. The resulting calibration curve showed a linear range from 50 μM to 1 mM (*R*^2^ = 0.9995), with a sensitivity of 14.13 μA cm^–2^ mM^–1^ and a limit of detection (S/N = 3) of 50 μM. The linear range obtained with the developed EFC allows glucose detection at the levels found in saliva and sweat.^39^ Nevertheless, the linear range of the sensor could be expanded by optimizing the enzyme and redox-mediator-loading on the electrodes^40^ or by using polymer coatings.^41^ Such enhancement of the linear range of the self-powered sensor would allow the detection of glucose in other physiological fluids such as the interstitial fluid, where the glucose concentration is usually higher than 3 mM.^39^

The long-term stability of the EFC is a crucial parameter for the development of self-powered applications, as a drift in the current output over time would lead to erroneous measurements and diagnosis. Therefore, the stability of the current output generated by the EFC was continuously measured for 5 days for a concentration of 6 mM glucose in phosphate buffer. As shown in Figure 5c, a stable current density of ca. 40 μA cm^–2^ was generated for 7 h which then dropped exponentially. The current generation curve was fitted to a simple exponential decay to obtain the half-life (*t*_1/2_) of the cell of 24 h (*R*^2^ = 0.99). GOx wired with osmium polymers to graphite electrodes has previously showed a 20% reduction in its activity after 12 h in phosphate-buffered saline (PBS).^42^ BOD may also desorb over time from the surface of the biocathode due to the physisorption-based immobilization of the enzyme, as previously reported.^28,31^ Likewise, the formation of hydrogen peroxide during the oxidation of glucose at the bioanode may further compromise the stability of the EFC and affect the catalytic activity of the biocathode over time.^43^

As shown in Figure 5c, continuous detection of glucose could be accurately performed only during the first 7 h of continuous operation. After 7 h, the current dropped exponentially, challenging the detection of any change in the glucose concentration. Therefore, for practical applications in the continuous mode, it is important to enhance the EFC stability over time.

As a proof-of-concept, the EFC was tested in artificial saliva spiked with increasing concentrations of glucose (Figure 5d). The EFC showed a sensitivity of 21.5 μA mM^–1^ cm^–2^ toward glucose in saliva, an increase of 52% compared to the sensitivity observed in buffer, which can be attributed to the higher conductivity of saliva compared to that of phosphate buffer (11.9 and 9.2 mS cm^–1^, respectively). Moreover, in saliva, a narrower linear range (0.75–2 mM) was observed as a result of the higher background current caused in saliva by its complex composition. The use of membranes or adjusting the enzyme/mediator ratio at the bioanode would help broaden the linear range of such device.^40,41^

A direct comparison with previously reported EFCs is difficult to perform due to the wide range of biological fluids and operation conditions tested, and the signal outputs measured, as shown in Table 1. Nevertheless, the EFC reported in this work shows a sensitivity toward glucose up to 2 orders of magnitude higher compared to other EFCs. The EFC reported in this study also shows a higher sensitivity toward glucose compared to the abiotic fuel cell on a PCB recently reported, and allows the detection of lower concentrations of glucose.^44^ While narrower compared to other studies reported, the linear range obtained with our EFC is within the concentration of glucose in saliva, thus demonstrating its applicability. Moreover, as opposed to previous EFCs reported, the PCB technology facilitates the development of EFC arrays to increase the current output,^44^ which can be easily integrated with electronic and microfluidic structures.^16^ These EFC arrays can be designed in such a way to meet the specific energy demands of the device, for example, data transmission, and consequently generate a self-sustained system.

**Table 1:** Comparison of EFCs for the Self-Powered Detection of Glucose^a^

anode	cathode	experimental conditions	output signal	sensitivity (μA mM^–1^ cm^–2^)	linear range (mM)	substrate	reference
GOx/Os/hPG	BOD/SB/hPG	PB 0.1 M, pH 7.4	current	14.13	0.05–1	PCB	this work
GOx/Os/hPG	BOD/SB/hPG	artificial saliva	current	21.50	0.75–2	PCB	this work
hPG	Pt	PB 0.1 M, pH 7.4	current	8.8	0.3–9	PCB	(44)
GOx/chit	activated carbon (air-breathing)	n.d.	current	0.02	1–5	paper	(45)
GOx/TTF	BOD	PB 1 M, pH 7.0	power	n.d.	1–25	paper	(46)
GDH/NADH/Vit K3	BOD (air-breathing )	PB 0.1 M, pH 7.0	power	0.004 μW cm^–2^ mM^–1^	0.5–10	needle	(47)
GOx/chit	activated carbon	artificial sweat	current	1.35 μA mM^–1^	0.1–5.5	paper	(48)
PQQ-GDH/Os	BOD/CNF	PBS 0.1 M, pH 7.4	power	n.d.	0.1–1	SPE	(49)

a Legend: glucose oxidase (GOx), highly porous gold (hPG), chitosan (chit), tetrathiafulvalene (TTF), glucose dehydrogenase (GDH), reduced nicotinamide adenine dinucleotide (NADH), glucose dehydrogenase dependent on pyrroloquinoline quinone (PQQ-GDH), phosphate buffer (PB), phosphate-buffered saline (PBS), printed circuit board (PCB), and screen-printed electrode (SPE). The sensitivity refers to the geometric area of the anode.

## 4 Conclusions

This study reports the first successful example of an EFC on a PCB for the self-powered detection of glucose in physiological fluids, such as saliva. The fuel cell exploits the enzymes BOD and GOx for the reduction of oxygen and the oxidation of glucose, respectively. The immobilization of enzymes onto the PCB Au electrodes led to low or no electrocatalytic activity, whereas an increase in the surface area of the electrodes by depositing a film of hPG resulted in improved current outputs. A blocking buffer was needed at the biocathode to prevent glucose oxidation by the hPG film where BOD is directly adsorbed. Therefore, the use of blocking agents on fuel cells opens up the possibility to protect the electrodes from common interferences. The resulting EFC showed a linear detection toward glucose up to 1 mM with a sensitivity of 14.13 μA mM^–1^ cm^–2^ and a limit of detection of 50 μM in phosphate buffer. The EFC was further tested in artificial saliva, obtaining a linear range of up to 2 mM glucose and a sensitivity of 21.5 μA mM^–1^ cm^–2^. The EFC can detect concentrations of glucose that are lower than those detected by the abiotic fuel cell previously reported by our group which, in addition to the greater sensitivity obtained with the use of enzymes, makes it an excellent candidate for sensing applications. Further optimization of the enzyme- and/or mediator-loading will be required to improve the linear range of the sensor and to increase the stability of the signal over time. Overall, the PCB technology opens up the possibility of a standardized manufacturing process for developing miniaturized EFCs for wearable and miniaturized diagnostic devices.

## Supporting Information Available

SEM images of the hPG electrodes; electrochemical characterisation of the BOD/hPG/Au cathode; electrochemical response to glucose of the hPG/Au electrode; and electrochemical characterisation of Os/hPG/Au and Os/Au electrodes in phosphate buffer (PDF)
