# Supplementary material for: Self-Powered Detection of Glucose by Enzymatic Glucose/Oxygen Fuel Cells on Printed Circuit Boards
Source: ACS Appl Mater Interfaces. 2021 May 26;13(23):26704–11. doi: 10.1021/acsami.1c02747 (PMC8735749; doi:10.1021/acsami.1c02747)
Supplement: Supplementary file 1 — am1c02747_si_001.pdf [file am1c02747_si_001.pdf]

Supporting Information

Self-powered Detection of Glucose by Enzymatic Glucose/Oxygen Fuel Cells on Printed Circuit Boards

Carla Gonzalez-Solino[1,2], Elena Bernalte[1,2]†, Clara Bayona Royo[1,2], Richard Bennett[3]††, Dónal Leech[3] and Mirella Di Lorenzo[1,2]*

[1]Department of Chemical Engineering, University of Bath, Bath BA2 7AY, UK

[2]Centre for Biosensors, Bioelectronics and Biodevices (C3Bio), University of Bath, Bath BA2 7AY, UK

[3]School of Chemistry & Ryan Institute, National University of Ireland Galway, University Road, Galway, H91 TK33, Ireland

*Corresponding author: mdl28@bath.ac.uk

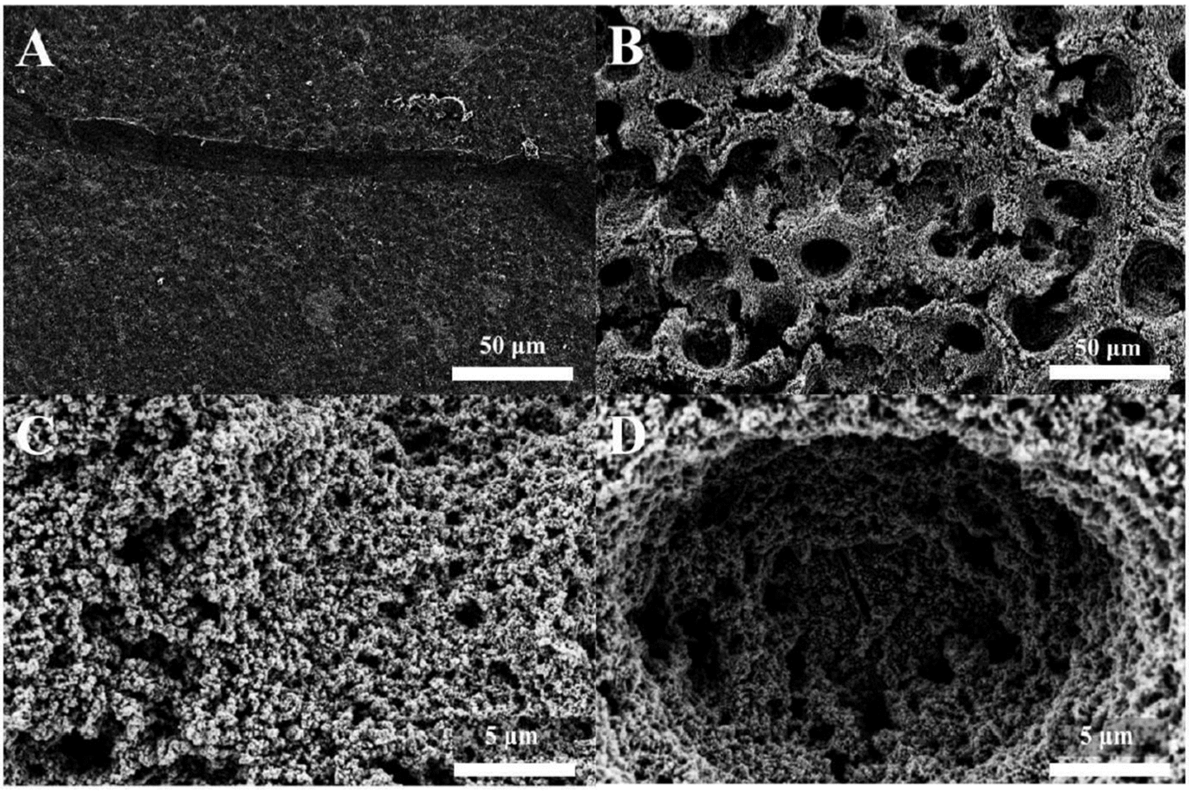

Figure S1. SEM images. (A) Au electrode on PCB at 500X magnification. (B) hPG/Au at 500X magnification. (C,D) hPG/Au electrode at 5k magnification.

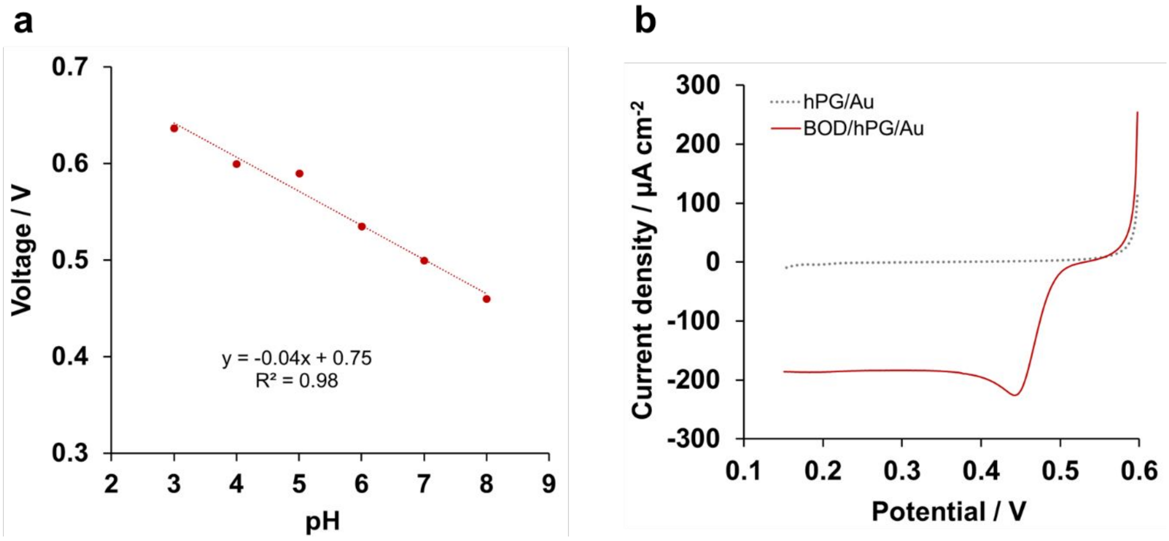

Figure S2. Characterisation of the biocathode BOD/hPG/Au: a) effect of pH on the ORR and b) ORR at the hPG electrodes with and without BOD.

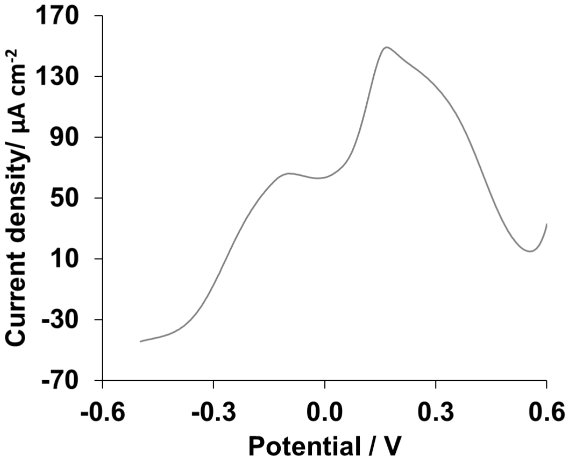

Figure S3. I-V response of the hPG/Au electrode in the presence of 6 mM glucose in phosphate buffer pH 7.4. Potentials referred to Ag/AgCl. Scan rate 5 mV s^{-1}.

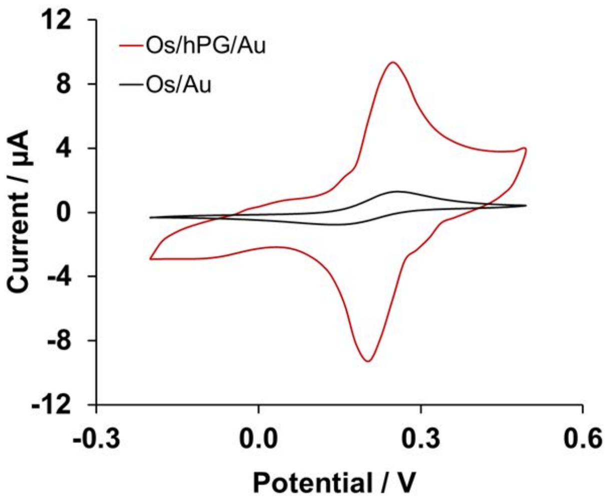

Figure S4. Comparison of Os/hPG/Au and Os/Au electrodes in phosphate buffer at a scan rate of 10 mV s^{-1}.
